# Clinico-epidemiological profile of children living with HIV/AIDS managed at Heal Africa Hospital, Goma, Democratic Republic of the Congo

**DOI:** 10.4314/ahs.v22i4.49

**Published:** 2022-12

**Authors:** Emil Mapera, Jean Pierre Fina, Joseph Body Mabiala, Lukanu Phillipe Ngwala, Doudou Nzaumvila

**Affiliations:** 1 Universite Protestante du Congo, Medecine de famille; 2 Universite de Kinshasa, Pediatrie; 3 Universite Protestante du Congo, Department of Family Medicine; 4 Sefako Makgotho Health Sciences University

**Keywords:** Central Africa, civil war, HIV/AIDS, pediatric, presentation

## Abstract

**Background:**

Conflict in the DRC led to a poor health care. HIV/AIDS in children remains one of the leading causes of pediatric morbidity and mortality.

**Methods:**

This cross-sectional study used a sample size of 238 files and aimed to determine the epidemiological profile of children living with HIV at Heal Hospital in 2015.

**Results:**

The age ranged from zero to fifteen, with a mean of 6.1 (±3.9) years. Records of PMTCT were noted in 12%. The mean birth weight was 3(±0.8) kg, most cases (88 percent) had normal vaginal delivery. Many of them (71 percent) were living with at least one parent. The majority of the children (92 percent) were from Goma, and 75 percent were diagnosed at WHO Stage 3. At least one episode of hospital admission was reported in 71 percent. Respiratory tract infections were the most common disease, and they were also the leading cause of death. Based on the CD4, which was the most cost-effective method of monitoring, there was an improvement in immunity at the last visit.

**Conclusion:**

This study pointed out the importance of PMTC and early management of children leaving with HIV/AIDS. Outreach would encourage voluntary HIV/AIDS testing for pregnant women in armed conflict zone.

## Introduction

In 2020, the human immunosuppression virus (HIV) infection, is still posing specific characteristics challenges among children and adolescents that influence how diagnosis, treatment and care are delivered. It needs care models that optimize the retention of children in the antiretroviral (ARV) program or treatment, to ensure viral load suppression and to overcome different life-course issues 1. To date, many countries continue to encounter substantial major different issues in preventing, diagnosing and treating children leaving HIV and acquired immunodeficiency syndrome (AIDS) as well as data collection reporting and analysis^1^,^2^. Pretty much across the board, these factors lead to the fact that only five percent of people living with HIV worldwide are children and adolescents younger than 15 years, but in 2019 they accounted for 14 percent of people dying from AIDS-related causes 1,3,4. This is making paediatric HIV to be a health priority today, as AIDS in children remains one of the leading causes of paediatric morbidity and mortality 5. It is estimated that 2.6 million children living with HIV, including 220,000 new infections and 150,000 paediatric HIV deaths 6. Almost 90 percent of children living with HIV and AIDS are in Africa 5, in the absence of any treatment, 52% die before their second birthday 7.

Sub-Saharan Africa accounted for 84% of new infections, 87% of HIV / AIDS deaths among children, and 11 million children orphaned due to HIV/AIDS 8. The Democratic Republic of Congo (DRC) has been experiencing armed conflict for the past 20 years. Added to this is the precarious health infrastructure and the quality of care as well as various diseases such as malaria, tuberculosis (TB), infection with HIV. Children are among the most affected by this situation 5. Children's HIV prevalence varies by country. Ogunbosi 9 reported 10% in Nigeria, and was documented at 6% in DRC 10,11. Regarding the average age of children infected with HIV, different data are found in the literature, ranging from 30 months to seven years 12–16. The most common infections in children infected with HIV are respiratory diseases, diarrheal diseases, malnutrition, anaemia and dermatitis 17. In the DRC, as in many other countries such as Morocco, diarrheal diseases followed by respiratory infections, malnutrition and dermatological diseases are the most common among children living with HIV 9,16.

HIV is a public health problem in general and in the city of Goma especially with the conflict context. Knowing the social environment of life of these children in addition to the epidemiological and clinical profile will improve the care of these children and reduce the morbidity and mortality caused by HIV / AIDS in the DRC in general and in the city of Goma in particular.

## Aim

This study aimed to determine the clinico-epidemiological profile of children living with HIV/AIDS managed at Heal Africa Hospital.

## Methods

The study was conducted at Heal Africa Hospital, located south of the city of Goma, in the municipality of Goma North Kivu Province. It is a 204-bed tertiary hospital with a specialized service for the care of children living with HIV/AIDS. The study population consisted of 600 children with HIV who attended the Heal Africa Hospital's wellness clinic. With a 95 percent confidence interval, a convenient sample size of 234 was calculated and rounded to a convenient number of 238 files. Children who met the following criteria were included in the study: HIV positive (confirmed) attending Heal Africa for more than six months; aged between 0 and 15 years. The data collection was carried out based on a pre-prepared questionnaire and data was collected from the registers and patient files. The following socio-epidemiological variables were collected: sex; age; provenance (place of residence of the child or his/her family); situation of parents (alive or dead); clinical history of the child. The following clinical variables were collected for each child at the clinical stage on admission, according to the WHO criteria for the care of children living with HIV/AIDS. We denote common pathologies as the various diseases for which the children consulted during the year. We analysed the data using SPSS Statistical Version 23.

## Ethical considerations

Before the start of the study, we obtained the approval of the Ethics Committee of the Protestant University in Congo (Reference: CEUPC0032) and permission from the Heal Africa management.

## Results

Table 1 indicates that children's age varied between zero and 15 years. The mean age was 6.1 (±3.9) years. We did not note an age difference with respect to sex. With regard to the provenance of the children surveyed, we found that 92 percent those recruited in the study came from the City of Goma. The study found that 71 percent of children living with HIV followed at Heal Africa Hospital live with at least one parent 29 percent who live either with a host family (22 percent) or in an orphanage (7 percent). Of the 238 children in our sample, the researchers found that 45 percent had both parents alive. The mean birth weight was 3 (±0.8) kg. A large majority of the children living with HIV followed at Heal Africa Hospital, namely 88%, were born from a normal delivery as illustrated in Table I. We found that only 5 (2%) did not have a BCG scar. Almost all 97 percent of the children observed had a normal psychomotor development. The history of blood transfusion was found in 5 percent of children. The history of hospitalization was found to be 71 percent of children. Of the 238 children in this study's sample, prevention of vertical transmission was found in only 12 percent of children living with HIV. Only 12 participants received a polymerase chain reaction (PCR) diagnosis in our study, 5 percent respectively at 2 months, 5 months and 7 months of age. The study showed that 75 percent of children living with HIV followed at Heal Africa Hospital were diagnosed at WHO Stage 3.

**Table 1:** Baseline characteristics

Characteristics	N	Percentage
Age		
0–4	109	45.8
5–9	86	36.1
10–15	43	18.1
Sex		
Female	119	50
Male	119	50
Birth weight		
≤ 2.6kg	31	13
≥ 2.6 kg	207	87
Family status		
Child lives with at least one parent	170	71
Child lives with a foster or host family	52	22
Child lives in an orphanage	16	7
Provenance		
Goma	233	98
Out of Goma	5	2
Parental status		
Both parents alive	108	45
One of the parents alive	67	28
Both parents deceased	46	19
Mode of delivery		
Eutocia	209	88
Dystocia	29	12
BCG scar		
Present	233	98
Absent	5	2
Psychomotor development		
Good	233	97
Bad	5	3
Blood transfusion		
At least once	13	5.5
Never	220	92
No data	5	3
History of hospitalization		
At least once	170	71
Never	68	29
PMTCT		
Yes	28	12
No	160	67
No data	50	21

Table 2 shows that 39(17 percent) suffered from respiratory tract infections more than once and it was the most common condition.

**Table 2:** Common conditions found among children living with HIV/AIDS

Diseases	Never n (%)	Once n (%)	More than once (%)
Anemia	232(97)	4(2)	2(1)
Dermatological lesions	153(64)	51(21)	34(15)
Diarrhea	195(82)	27(11)	16(7)
Lymphadenopathy	227(95)	10(4)	1(0)
Malnutrition	223(94)	9(4)	6(2)
Otitis	191(80)	28(12)	19(9)
Prolonged fever	168(71)	53(22)	17(7)
Respiratory tract infections	138(58)	61(26)	39(17)
Other pathologies		15(6)	8(4)

Table 3 indicates that 13(4.5 percent) cases of death with respiratory tract infections being the lead cause 5 (38.5 percent).

**Table 3:** Causes of death among children leaving with HIV (n=13)

Cause of death	N
Respiratory tract infections	5(38.5)
Malnutrition	3(23.1)
Anaemia	2(15.4)
Acute gastroenteritis	2(15.4)
Malaria	1(7.6)

Total	13

**Figure 1:**
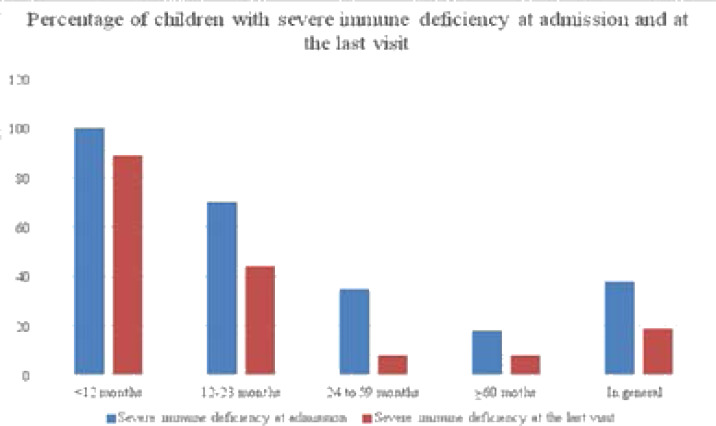
Percentage of severe immune deficiency

At the time this study was conducted, the viral load test was still very expensive and unaffordable for many, reason why at heal Africa Hospital, the CD4 count was used to evaluate progress. Figure 1 shows that there was an improvement in immunity at the last visit compared to when the patient was admitted to the wellness program.

## Discussion

Our findings showed that children at Heal Africa Hospital were diagnosed and or began ARV treatment relatively late, at around 6 years of age. The same pattern has been observed in some African countries 12, also in other part of the DRC 16. In other countries, children commence the treatment earlier, Bedri 14 reported 3.8 years in Ethiopia, and Kadi 18 in Morocco noted 2.5 years. The cause of this difference has to be found within the socioeconomics difficulties of the DRC, while in other countries the paediatric HIV diagnostic is done by PCR, at the time this study was completed, this test was unaffordable for the communities' members around Heal Africa Hospital. Most of the children were diagnosed at later stage of the diseases with opportunistic infections; the majority were already in WHO stage 3. In addition, the low PMTCT coverage in the area has resulted in many missed care opportunities of children born to HIV positive mothers, therefore the child will only be screened if presenting opportunistic with infections later in their live. In this study, we did not find gender-related disparities; both sexes reached similar proportions as found by M'Pemba 15. By contrast, Mwadianvita 16, Ogunbosi 9 and Soubeira 19 found in their series a predominance on the part of male children. This situation is dependent on the sex balance which does not show great disparities, according to the DS RDC 2013–2014.

Ninety-two percent of children live in or come from urban areas, compared to eight percent from rural areas. This was noted by some authors who documented the living areas in their studies. 14, 15. In addition to the fact that Heal Africa Hospital is situated in urban area, the current situation of the war in the DRC has contributed to the migration of the population to safer (urban) areas. The DRC suffers from many economic problems that social welfare structure does not function properly to the extent of not existing at all in the parts affected by the war. Our findings showed seven percent live in orphanage. This ssituation contrasts with that found in some countries such as Morocco 18, Burundi 20 and Congo Brazzaville 15 where this proportion was 44 percent, 39.3 percent and 39 percent respectively. Even though supporting structures like orphanage could have been available, social cultural practice should have prevailed in such way that an orphans will be adopted by a family member than to be sent in an orphanage.

Our study revealed that 91.2 percent of the participating children had HIV-positive mothers. What underlines that in more than 90 percent of instances that transmission was vertical. This result confirms the predominance of vertical transmission in children, in keeping with WHO experts 21. However, Soubeiga 19 recorded 80% of vertical transmission with PMTC coverage of 82 percent while we had 12 % of children who benefited from PMTCT programme. Could this suggest a vulnerability of the medical system in a post-conflict context? This would contrast with the fact that 98% of children had a BCG scar that demonstrated the accessibility l o f these children to the Expanded Program of Immunization (EPI) at birth. This highlights the weaknesses that persist in the adherence of PMTCT mothers to antenatal consultations (ANC) in our midst where stigma and discrimination persist because of low community involvement 22. Another explanation could be that PMTCT, unlike EPI, is a relatively new programme, thus contemporary with the armed conflict. One could see the difficulties of developing the PMTCT programme under such conditions.

In comparison to other studies that recorded 28 percent in Congo Brazzaville 15, or 69 percent in India 12 and Lubumbashi 16, the history of blood transfusion was found in 13 patients, or 5.5 percent. This difference can be explained by the fact that the children in our study are all on ARV's and adhere to treatment programs. This has the benefit of immune regeneration and hence the separation of episodes of disease. This has the consequence of immune restoration and therefore the spacing of disease episodes.

A comparison of the proportions of cases of severe immunodeficiency at admission and at the last visit showed a decrease ranging from 38 percent at admission to 19 percent at the last visit. We also found that younger children are more affected by severe immune deficiency. In our study, 38 percent of children had severe immune-suppression and younger children were more affected by severe immune deficiency. This was lower than Ogunbosi's result 9 that found a severe deficit in 57.8 percent of children living with HIV. Only 5 percent of children were able to have a diagnostic confirmation of the PCR. This trend has been observed by other authors 23 and raises the crucial question of the accessibility of early diagnosis in a context of limited resources with low laboratory coverage, as is the case in our study context. This study also showed that 75 percent of the children were admitted at clinical stage 3 according to WHO. Our results coincide with those found in Nigeria 9, but contrast with data from a study conducted by Mwadiamvita 16 in Lubumbashi and Arjit 24 that found respectively 24.3 percent and 40 percent of children had been admitted at advanced stages of the disease. This could be explained by the fact that the diagnosis of HIV/AIDS in children in our environment has been long delayed.

Respiratory diseases were the most common opportunistic infections. Our results are compatible with the results of other countries 15,20,23. Episodic diarrheal diseases were found in a proportion of 17 percent, a little higher compared to the result of the Malian study 23. ENT diseases mainly otitis, were observed in similar proportion in India 8 and a little less by Traoré 23 in Mali with respectively 12% and 5% of cases of otitis in their studies.

The reasons for hospitalization were respiratory opportunistic infections, followed by unexplained prolonged fever, malnutrition, anaemia and diarrhoeal diseases. Our findings are consistent with others 12,20,21. The death rate among HIV-positive children followed in Heal Africa was 4.5 per cent. Respiratory diseases were the leading causes of death in our study, followed by malnutrition. The same was reported by M'Pemba 15. Ndondoki 25 findings indicated diarrheal diseases as leading cause of deaths whereas Floquet 26 reported of more cases of malnutrition as the leading cause of death. This diversity may be attributed to the environmental context of life but also to the viral strain, mainly in East Africa, where HIV 2 has low virulence.

## Conclusion

The present study emphasised the significance of PMTC on the need for way earlier management of children living with HIV / AIDS. All of these have been made difficult by the armed conflict in the area. An adapted and appropriate outreach program could have made a difference in supporting parents or caregivers of children living with HIV/AIDS to have access to care.

## Recommendations

A number of strategies could improve the quality of life for children living with HIV. Firstly, new outreach strategies that promote community participation in HIV/AIDS voluntary testing for pregnant women during antenatal consultation sessions. Secondly, there should be an improvement in accessibility to PMTCT service provision in the province in general and in Goma itself by targeting the most vulnerable communities in civil war circumstances. Laboratory coverage should be increased as well as the early detection of children living with HIV by improving access to sick children in order to improve their quality of life.
